# Characteristics of users of a tailored, interactive website for parents and its impact on adolescent vaccination attitudes and uptake

**DOI:** 10.1186/s13104-015-1721-8

**Published:** 2015-12-01

**Authors:** Amanda F. Dempsey, Julie Maertens, Brenda Beaty, Sean T. O’Leary

**Affiliations:** Adult and Child Center for Outcomes Research and Dissemination Science (ACCORDS), 13199 East Montview Blvd, Suite 300, Aurora, CO 80045 USA

**Keywords:** Vaccine, Adolescent, Human papillomavirus, Meningococcal, Pertussis, Influenza

## Abstract

**Background:**

We examined the characteristics of parents using an iPad-based intervention about vaccines, and its impact on vaccination attitudes and behavior.

**Methods:**

Interventions were implemented in three primary care clinics from June 2012–September 2013. Baseline and follow up surveys assessed vaccination attitudes and intentions. Medical records were used to examine adolescent vaccine uptake.

**Results:**

During the study, 42 parents viewed tailored educational content. Users were generally positive about vaccines, though one out of five worried that vaccines caused more harm than good. Among the 16 parents completing the post-intervention survey, there was a slightly higher, non-statistically significant, mean vaccination intention after viewing the website than prior to viewing it for three of the four adolescent vaccines (all except tetanus–diphtheria–acellular pertussis). Using the intervention did not increase the likelihood of adolescent vaccination.

**Conclusions:**

Providing educational material via iPads in clinic waiting rooms does not appear to be an effective strategy for engaging parents about vaccines. Overall, parents’ interaction with TeenVaxScene was low, and had little impact on their vaccination attitudes and beliefs. However, use of TeenVaxScene did not appear to *worsen* parents’ attitudes about vaccines. New and creative ideas for engaging parents to use such educational materials are needed.

## Background

Recognizing that adolescence is a time of major risk for several important vaccine preventable diseases, [1] the Advisory Committee on Immunization Practices (ACIP) established the “adolescent platform” of vaccines in 2005. This platform includes the human papillomavirus (HPV), tetanus–acellular pertussis–diphtheria (Tdap), influenza, and meningococcal (MCV) vaccines. The ACIP recommends Influenza vaccine to all adolescents yearly and the remaining three vaccines as part of routine preventive health care for 11–12 year olds, with catch up vaccination recommended for older adolescents who have not yet been vaccinated [2].

Healthy people 2020 established a goal coverage level of 80 % among adolescents for each of these four vaccines [3]. However, national studies indicate that while coverage among adolescents for Tdap and MCV vaccines have reached or nearly reached this level (86 and 78 % respectively for 13–17 year olds as of 2013), utilization of HPV and Influenza vaccines is lagging significantly [4, 5].

Barriers to high adolescent vaccine coverage include parents being unaware of adolescents’ risk for vaccine preventable diseases, and negative parental attitudes about the safety and necessity of adolescent vaccines [6–12]. While these barriers must be overcome for high vaccination levels to be achieved, there is a paucity of research on interventions to do this. Intervention development has been hindered by the difficulty in finding time during clinical encounters to adequately address parents’ vaccine-related questions, and the wide variety of parents’ vaccination beliefs and informational needs.

One promising strategy that may begin to address this problem is that of “tailored messaging.” Tailored messaging is based on the principal that when information is provided to the user in a way that feels *personally* relevant, that information will be more impactful and thus more likely to be acted upon. A large body of evidence supports the effectiveness of tailored messaging in improving compliance with a variety of preventive health behaviors, [13, 14] and our prior research supports its potential to increase levels of adolescent vaccination [15, 16].

Our group recently developed an educational website that provides individually-tailored information to parents of adolescent children about the four vaccines in the adolescent platform. To our knowledge this website, called TeenVaxScene, is the first tailored messaging intervention related to adolescent vaccination to be developed. The goals of this study were to better understand the characteristics of parents who used the TeenVaxScene website (described in detail previously [17]) when provided in outpatient pediatric clinic waiting rooms via iPads, whether using TeenVaxScene impacted parental attitudes and decision making about adolescent vaccination, and ultimately if use of TeenVaxScene impacted adolescent vaccination.

## Results

### Pre-intervention survey results

#### Demographics

During the study period (July 2012–Sept 2013), there were 6749 adolescent patients seen in the three practices. As reported elsewhere, relatively few parents interacted with the intervention despite the implementation of several different types of recruitment strategies [17]. Table 1 shows the demographic characteristics of the 54 parents who used TeenVaxScene during the study period, and includes the characteristics of these parents’ 74 adolescent children. Participants were distributed equally across the three clinics (data not shown). Most were highly-educated, white females. A notably high proportion of parents, as well as their adolescent children, had personally experienced influenza in the past (Table 1). Among parents, 74 and 68 %, respectively, indicated that they themselves had previously received Tdap and Flu vaccines.

**Table 1:** Demographic characteristics of TeenVaxScene users and their adolescent children

	% (n)
Parent characteristics, n = 54	
Age in years (range, SD)	41.8 (18–54)
Female gender	66 (31)
Marital status	
Single	17 (8)
Married/partnership	76 (36)
Divorced/widowed	7 (3)
Hispanic	10 (5)
Race	
White	87 (41)
Black	0
All other choices	13 (8)
Education	
<High school	2 (1)
High school graduate	11 (5)
Some college	86 (41)
Prior personal experience with…	
Flu	83 (45)
Tetanus	0
Pertussis	22 (12)
Meningitis	6 (3)
Positive HPV test	7 (4)
Genital warts	4 (2)
Abnormal pap smear	30 (16)
Cervical cancer	9 (5)
None of the above	13 (7)
Prior experience of illness in their adolescent with…	
Flu	63 (34)
Tetanus	0
Pertussis	13 (7)
Meningitis	2 (1)
HPV-related health problems	0
None of the above	31 (17)
Adolescent characteristics, n = 74^a^	
Adolescent’s age in years (range)	13.6 (11–17)
Female gender	51 (36)
Adolescent’s health	
Very good	69 (51)
Good	30 (22)
Fair	1 (1)
Poor	0
Medical conditions in adolescent	
Lung problems	15 (11)
Neurologic problems	4 (3)
Other chronic problem	4 (3)
Allergy to eggs	0
None of the above	78 (58)
Aware that there are vaccines recommended for this adolescent	84 (62)

^a^Parents could answer questions about as many adolescents as they had in their family

#### Prior receipt of adolescent vaccines

In the pre-intervention survey, Tdap and Influenza vaccines were reported to have been received by the participants’ adolescent children in 77 and 64 % of cases, respectively. Prior receipt of MCV and HPV vaccines was notably lower at 53 and 56 %, respectively. Among the 39 adolescents whose parents reported prior HPV vaccination, 29 (59 %) reported that three doses had been received, 15 (28 %) indicated that their adolescent had not yet gotten three doses, but planned to do so, and one parent (3 %) indicated their adolescent had not yet gotten three doses and they did not plan to allow any more.

#### Attitudes about vaccines

Even before viewing the TeenVaxScene website, parents reported relatively high perceived effectiveness of the adolescent vaccines. Parents rated the Tdap and MCV vaccines as “very effective” or “effective” 73 % of the time, while Influenza and Tdap vaccines were rated this highly 69 and 65 % of the time, respectively. More than half of parents (55 %) indicated they wanted more information about how vaccines work, and 58 % indicated they wanted more information about vaccine safety.

Table 2 shows results of the pre-intervention survey regarding parents’ attitudes about vaccines in general, and for each vaccine the parent reported their adolescent had not yet received. As in other studies, [6, 20] a high proportion (69 %) indicated that a doctor’s recommendation was one of the most important factors in deciding about vaccines. However, a number of negative attitudes about vaccines were also identified. For example, one out of five parents worried that vaccines in general caused more harm than good. Moreover, a substantial minority of parents believed that vaccines in general might cause long term health problems for their child (15 %), that the HPV vaccine might make their adolescent think it was OK to have sex (23 %), and that the Influenza vaccine could cause the flu (34 %).

**Table 2:** Attitudes about vaccines among TeenVaxScene users

Statement	% (n) agree or strongly agree
General attitudes, n = 65	
I think vaccines may cause short-term problems (example: fever or pain)	42 (27)
I think vaccines may cause serious long term health problems for [child’s name]	15 (10)
Many of my friends are not getting their children vaccinated	9 (6)
Having [child’s name]’s doctor recommend a vaccine is one of the most important factors in deciding to get [him/her] vaccinated	69 (45)
I worry that vaccines may cause more health problems for [child’s name] than benefit	20 (13)
HPV vaccine, n = 42	
I worry that [child’s name] may be at risk for genital warts, [(female): cervical cancer or other cancers related to HPV] OR [(male): cancers related to HPV, or may transmit the infection to others]	17 (7)
I worry that the HPV vaccine will give [child’s name] the HPV virus	4 (2)
I worry that [child’s name] will have reproductive problems if he/she gets the HPV vaccine	7 (3)
I worry that the HPV vaccine will cause immune problems for [child’s name]	7 (3)
I worry that if [[child’s name] got the HPV vaccine, then [he/she] would get neurologic problems] or [his/her neurologic problems would worsen]	9 (4)
I don’t think [my son] would get much benefit from getting the HPV vaccine^a^	10 (2)
I think that giving the HPV vaccine to [child’s name] may make [him or her] think it is OK to have sex^b^	23 (9)
I think [child’s name] is too young to get a vaccine for a sexually transmitted infection like HPV^b^	23 (9)
I am concerned that the HPV vaccine costs more than I can pay^b^	13 (5)
The HPV vaccine is so new that I want to wait a while before deciding if [child’s name] should get it^c^	27 (10)
I need more information about the HPV vaccine before deciding whether or not to give [child’s name] the vaccine^c^	27 (10)
Many of my friends are against giving the HPV vaccine to adolescents^c^	7 (3)
I would regret it if [child’s name] did not get the vaccine and later got a health problem related to HPV^c^	68 (25)
Tdap vaccine, n = 14	
I worry that [child’s name] may get tetanus 1 day	35 (5)
I worry that [child’s name] may be exposed to pertussis (whooping cough)	43 (6)
I worry that the Tdap vaccine is not safe for [child’s name]	21 (3)
I think that giving [child’s name] the Tdap vaccine is more harmful to [him/her] than not giving it	21 (3)
I would have [child’s name] get the Tdap vaccine because it is required by [his/her] school	64 (9)
Meningococcal vaccine, n = 22	
I worry that [child’s name] may be at risk for getting meningitis	14 (3)
I worry that the meningococcal vaccine is not safe for [child’s name]	14 (3)
I think that giving [child’s name] the meningococcal vaccine is more harmful to [him/her] than not giving it	10 (2)
I would have [child’s name] get the meningococcal vaccine because it is required by [his/her] school	55 (12)
I worry that if [child’s name] got the meningococcal vaccine, then [[he/she] would get neurologic problems] or [his/her neurologic problems would worsen]	10 (2)
Flu vaccine, n = 18	
I worry that [child’s name] may be at risk for getting the flu	24 (6)
I think the flu vaccine can cause [child’s name] to get the flu	34 (6)
I worry that if [child’s name]got the flu vaccine [he/she would get neurologic problems or his/her neurologic problems would worsen]	22 (4)
I don’t think the flu vaccine is safe	16 (3)
It is a hassle to get this vaccine every year	16 (3)
I think it is better for [child’s name] to have the flu than get the flu vaccine	11 (2)

The number of respondents changes for each section as parents were only asked about vaccines they reported their adolescent had not received previously. All parents received the general vaccination attitudes questions. Bracketed responses denote tailoring of the question to the child’s gender and/or neurologic status

^a^Only respondents with sons were provided with this question, n = 19

^b^Only 40 respondents answered this question

^c^Only 37 respondents answered this question

### Immediate impact of TeenVaxScene on vaccination attitudes

**Fig 1:**
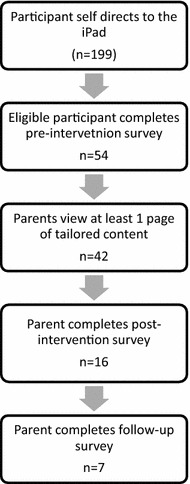
Participant flow through the various components of the TeenVaxScene intervention

Among the 54 parents who answered questions in the pre-intervention survey, 42 viewed at least one page of tailored content (Fig. 1). Of these, 16 (38 %) provided answers to the post-intervention survey to allow for assessment of the immediate impact of the TeenVaxScene website on vaccination intentions. As shown in Table 3, for three of the four vaccines (all except Tdap) there was a slightly higher mean vaccination intention after viewing the website than prior to viewing it. However, these results were not statistically significantly for any vaccine.

**Table 3:** Reported mean likelihood of having adolescent vaccinated before and after viewing TeenVaxScene

Vaccine	N for analysis	Pre intervention mean	Post intervention mean	p value
HPV	16	6.025	6.69	0.244
Tdap	4	8.00	8.00	n/a
Meningococcal	10	6.30	6.80	0.177
Flu	7	3.42	4.00	0.532

Analysis limited to participants who had data for both the pre-intervention and post-intervention surveys. The number of respondents varies by vaccine as parents were only asked to report on those vaccines they indicated their adolescent had not yet received

### Longer term impact of TeenVaxScene

#### Impact on vaccination attitudes

Among the 16 parents who completed the post-intervention survey, 8 (50 %) completed the follow up survey administered 3 months after viewing TeenVaxScene. As shown in Table 4, none of the parents indicated in the follow-up survey that TeenVaxScene made them *less likely* to have their adolescent vaccinated. In fact, in several cases, the website actually *increased* planned vaccination behavior (20–67 % of cases, depending on the vaccine, Table 4). This possible trend towards more positive vaccination intentions did not appear to be moderated by changes in perceived effectiveness of the vaccines or general vaccination attitudes, as neither of these measures were substantially more positive when comparing responses in the pre-intervention and follow up surveys for these parents (data not shown).

**Table 4:** Impact of TeenVaxScene on planned vaccination behavior

Vaccine	N for analysis	More likely to get	Don’t know	Less likely to get
HPV	5	1	4	0
Tdap	3	2	1	0
Meningococcal	5	1	4	0
Flu	3	2	1	0

Data reported for parents who responded to the follow up survey 2 months after viewing TeenVaxScene, and indicated that their adolescent had still not received the given vaccine. The number of respondents varies by vaccine as parents were only asked to report on those vaccines they indicated their adolescent had not yet received

#### Impact on vaccine uptake

There were 37 adolescent children whose parents used TeenVaxScene that had vaccination records available from the practice. Viewing TeenVaxScene did not appear to increase the likelihood that these adolescents got vaccinated on the day of their appointment (Table 5), or in the 3 months after the appointment (data not shown), when compared to adolescent patients seen in the clinic during the study period whose parents had not used TeenVaxScene, though this analysis was hindered by a small sample size.

**Table 5:** Impact of TeenVaxScene on vaccine uptake

	Utilizers	Non-utilizers	p value
Tdap	25 % (1/4)	35 % (695/1965)	0.99***
Flu	8 % (3/37)	25 % (1690/6712)	0.02
MCV	20 % (1/5)	38 % (823/2162)	0.66***
HPV	17 % (4/23)	19 % (969/5004)	0.99***

Among those eligible for the vaccine at the time of the visit, how many got a vaccine that day

*Utilizers* adolescents seen in the clinic during the study period whose parent used TeenVaxScene; *Non-utilizers* adolescents seen in the clinic during the study period whose parent did not use TeenVaxScene

*** Fisher’s exact test

## Discussion

In this study we implemented and assessed an educational website for parents called TeenVaxScene that provided individually-tailored educational material about the adolescent vaccination platform. The goal of this study was to assess the characteristics of those who used this website, and its impact on parental vaccination attitudes, decision-making and behavior. Overall, utilization of the website was low and appeared to have minimal impact on these outcomes. Thus, in a sense, our study could be considered a ‘negative study’ in that, despite extensive efforts to engage parents to participate in the website (reported elsewhere, [17]), very few actually did. This negative finding is important because iPad-based interventions have been successfully used in primary care settings for other health issues, and many researchers are currently examining the utility of web-based interventions to improve adolescent health [21–33]. Our study suggests that when it comes to educating parents about adolescent vaccines, passively placing an iPad or a kiosk in a waiting room is not likely to engage a high proportion of parents. Further study is needed to better understand how to actively engage parents with this type of information.

A major limitation to drawing conclusions from this study was its small sample size. Although the clinics where the intervention was in place saw ~6700 adolescents during the study period, only 54 parents completed the baseline survey portion of the intervention, and only 42 parents viewed tailored educational materials. As described previously [17], a number of different and increasingly intensive recruitment strategies were used to try and engage parents with the intervention. Despite this, the level of engagement with the intervention was disappointingly low, and significantly less that the level our study was powered for (n = 300). Future research will be needed to understand other ways such an intervention might be better disseminated—for example through pre-visit communication (i.e., emailed appointment reminders). However, it is important to note that when it comes to vaccination, interventions with even small impacts, or used by only small subset of the population can be potentially useful. This is because, with millions of adolescents being recommended for vaccines each year, interventions that provide even incremental improvements in vaccine uptake can lead to several thousands more teens getting their recommended vaccines. Thus, a potentially important finding from our study is the non-statistically-significant trend for increased vaccination intentions after viewing the TeenVaxScene website. It is possible that with a larger sample size (for example if TeenVaxScene was available to the general public), improvements in vaccination intention following use of TeenVaxScene would be statistically significant, which would be in keeping with our prior studies [15, 16]. However, an important caveat is that because the intervention was passively delivered, there was likely bias in the those who chose to use it—the intervention may have been more compelling to those who were inherently more interested in, and/or more willing to vaccinate, or alternatively, to those with strong convictions against vaccination that would be unlikely to be swayed by such an intervention.

Overall the attitudes of parents using TeenVaxScene were generally positive—most parents perceived vaccines as highly effective, and most of their adolescents had received at least one recommended adolescent vaccine. However, there were notable exceptions. For example, one out of five parents agreed or strongly agreed that “vaccines may cause more health problems than benefit” and more than one out of ten parents agreed or strongly agreed that any given adolescent vaccine was “unsafe.” The combination of prevalent negative attitudes about vaccines combined with high levels of reported vaccine uptake suggest that our intervention may appeal most to the “fence sitter” parent—that is, a parent who has enough concerns about vaccines that they are at risk of becoming a vaccine refuser in the future, though they are currently (mostly) following the recommend vaccination schedule [34]. Vaccine hesitancy is recognized as a spectrum of beliefs ranging from refusal of all vaccines to acceptance of all vaccines though with significant trepidation [35]. Prior research suggests that the “fence sitter” group of parents may be the most amenable to educational interventions such as TeenVaxScene as these groups have generally positive attitudes about vaccines, but need significant reassurance [34, 35]. Time constraints during clinical encounters have been reported by clinicians as a major barrier to addressing vaccine hesitancy among parents [10, 36–38]. Providing an educational resource such as TeenVaxScene prior to a clinical encounter may be an effective way to alleviate the concerns of “fence sitter” parents without adding significant time to the clinician–patient conversation. Our group currently has studies that are exploring this hypothesis directly.

Most of the users of TeenVaxScene in this study were educated, white females. This is not entirely surprising since mothers are known to be the parent most likely to take their child to the doctor, [39] and white was the predominant race among patients attending the clinical sites where the intervention was located. However, it was notable that a majority (86 %) of TeenVaxScene users had at least some college education, which differs from the demographics of the parent population at these clinics. Past research suggests that in the US vaccine hesitancy may be more common among more highly educated parents [40]. Thus, even though the level of utilization of TeenVaxScene was low overall, our results could be consistent with the notion that the intervention reached the “type” of parents (e.g., well-educated fence-sitters) who are most likely to have vaccine-related questions, concerns or informational needs.

A very important thing to consider in any intervention study is unanticipated negative impacts resulting from the intervention. Vaccine hesitant parents frequently report that available vaccine information is biased, emphasizing only the benefits to vaccination while minimizing or ignoring any side effects [41]. TeenVaxScene was designed with this in mind and special attention was given to trying to provide accurate, equal amounts of information about the pros and cons (risk of anaphylaxis, and short term side effects like fever, sore arm, headache, etc.) to vaccinating. Given this, one might hypothesize that providing extensive vaccine-related information to parents could actually *increase* their concerns about vaccines [42]. However, with the caveat that any results from our study should be considered cautiously given the small sample size, our very preliminary results do not suggest that TeenVaxScene causes negative vaccination beliefs and attitudes. For example, no parent reported in the follow up survey that they were *less* likely to get a vaccine for their adolescent after viewing TeenVaxScene (and several reported they were more likely to get vaccinated), and there was no measurable negative impact on perceived vaccine effectiveness, or general attitudes about vaccination.

In addition to the small sample size, there are other important limitations to this study that need to be considered. This pilot study was performed in a single geographic area, with minimal heterogeneity of the study population which limits the generalizability of the results. Also, there are known to be culturally-specific vaccination attitudes and beliefs [43–46] which would not likely be addressed by TeenVaxScene as it was designed for the general public. Finally, our website was available only in English and only on an iPad, and was therefore not likely useful for non-English speakers and those who were not familiar with how to use an iPad. Our sample could therefore have been biased towards more highly educated, computer savvy individuals, which may or may not be the group for which the information in the intervention is most useful.

## Conclusions

We developed a website called TeenVaxScene that provides individually-customized information about adolescent vaccination to parents [17]. We found that the majority of users were highly educated females, consistent with prior studies suggesting that this population is most likely to have significant concerns or questions about vaccines. Overall, parents’ interaction with TeenVaxScene was low, and had little impact on their vaccination attitudes, beliefs or behaviors, though there was a non-statistically significant trend for improved vaccination intentions following use of the website. Importantly, use of TeenVaxScene did not appear to *worsen* parents’ attitudes about vaccines. Larger studies are needed to understand the impact of this intervention on parents’ vaccine-related attitudes and beliefs, and how these are related to vaccine utilization among adolescents.

## Methods

### The TeenVaxScene website

The content and structure of “TeenVaxScene” has been described in detail elsewhere [17]. Briefly, this website creates individually-tailored content for parents that accounts for each person’s unique beliefs and experiences related to adolescent Tdap, HPV, Influenza and MCV vaccination. The goal of TeenVaxScene is to provide information to parents to help address their unique concerns or questions they may have about adolescent vaccines, and ultimately to increase adolescent vaccination uptake.

### Study setting

The TeenVaxScene website was provided via a kiosk or moveable iPad to parents of adolescents (ages 11–17 years) in the clinic waiting rooms of three primary care pediatric clinics in the Denver metro area. Practices A and B had patient populations that were primarily privately insured whereas nearly half (45 %) of the patients seen in Practice C were insured by Medicaid. Caucasian was the predominant race of adolescent patients at all three sites. All study activities were approved by the Colorado Multiple Institution Review Board (COMIRB) affiliated with the University of Colorado Denver.

### Study design

This study included a pre-/post-intervention design comparing the attitudes and vaccination intentions of parents before and after using the interventional website, and also a comparative cohort design whereby the vaccination status of children whose parents used the interventional website were compared to those age-eligible children seen in the clinic during the study period whose parents did not use the website.

### Study flow

Figure 1 depicts the various components of the TeenVaxScene intervention, and the number of participants at each step. Interested parents self-directed to the kiosk and used TeenVaxScene prior to their adolescent’s scheduled appointment (n = 199). After parents provided online informed consent for their participation in the study and approval to access their adolescent’s vaccination records, parents completed a brief “pre-intervention” survey that collected demographic data about the parent and adolescent, and assessed initial attitudes about vaccines and vaccination intentions (n = 54). As described previously, [17] the majority of parents were offered a $10 incentive for completing the pre-intervention survey. An internal “tailoring engine” [18] used the data from the pre-intervention survey to generate a series of webpages that were specific to each parent. After viewing the tailored content (n = 42), parents were asked (but not required) to take a brief “post-intervention” survey that re-assessed vaccination intention (n = 16), and also queried about their willingness to receive a “follow up” survey (with an additional $10 incentive) via postal mail 3 months after the date they used TeenVaxScene. The follow up survey (n = 8) re-assessed general vaccination attitudes, and collected data on the perceived usefulness of the intervention for making vaccination decisions. Up to three contact attempts were made via postal mail for the follow up survey. All survey materials are available from the authors upon request.

### Outcome measures

Attitudes about vaccines in general, and each vaccine specifically, were assessed by measuring agreement with a series statements using a 5-point Likert scale ranging from “strongly agree” to “strongly disagree.” All parents received statements regarding their attitudes about vaccines in general, but vaccine-specific statements were provided only to those parents reporting that their adolescent had not yet received that particular vaccine. Due to the small sample size, categorical Likert responses were grouped for each question to improve statistical power. For the attitude assessment, responses were grouped as “strongly agree/agree” versus “neutral/disagree/strongly disagree;” for the assessment of vaccine effectiveness responses were grouped as “very effective/effective” versus “unsure/somewhat ineffective/very ineffective.” Vaccination intention was measured using a previously-described 11-point scale, where mean vaccination was calculated across participants for each vaccine, and higher values represented more positive vaccination intentions [19].

Vaccination status was assessed by linking unique identifying information (i.e., name and birthdate) of the children of parent participants who self-elected to use the website to the child’s medical records at that practice and to the state of Colorado’s Immunization Information system. The comparison group was all age-eligible adolescents attending the clinic during the study period whose parents did not appear to have used the intervention website. Based on what we had initially estimated would be the sample size of those using the intervention (n = 300) we had powered our study to be able to detect seven percentage point differences between children of parents who did and did not use the intervention. However, the extremely poor uptake of the intervention meant our ability to find statistically significant differences between groups was severely limited, even if the percentage point variation between groups was large.

### Data analysis

Descriptive statistics were generated for all demographic variables. Two-sided, paired Student t tests were used to compare mean vaccination intentions between the pre- and post-intervention surveys. Fisher’s exact tests were used to compare vaccination attitudes between the pre-intervention and follow-up surveys. A p value of ≤0.05 was considered statistically significant. All analyses were performed in either SAS 9.2 (Cary, NC) or STATA12 (StataCorp LP).

## Abbreviations

Tdap: tetanus–diphtheria–acellular pertussis
ACIP: Advisory Committee on Immunization Practices
HPV: human papillomavirus
MCV: meningococcal conjugate vaccine
COMIRB: Colorado Multiple Institutional Review Board
